# Fifty years of Brazilian Dental Materials Group: scientific contributions of dental materials field evaluated by systematic review

**DOI:** 10.1590/1678-775720150560

**Published:** 2016

**Authors:** Wellington Luiz de Oliveira ROSA, Tiago Machado SILVA, Giana da Silveira LIMA, Adriana Fernandes SILVA, Evandro PIVA

**Affiliations:** 1 - Universidade Federal de Pelotas (UFPel), Faculdade de Odontologia, Pelotas, RS, Brasil.; 2 - Universidade Federal de Pelotas (UFPel), Faculdade de Odontologia, Departamento de Odontologia Restauradora, Pelotas, RS, Brasil.

**Keywords:** Dental materials, Dentistry, Innovation, History, Review

## Abstract

**Objective:**

A systematic review was conducted to analyze Brazilian scientific and technological production related to the dental materials field over the past 50 years.

**Material and Methods:**

This study followed the Preferred Reporting Items for Systematic Reviews and Meta-Analysis (Prisma) statement. Searches were performed until December 2014 in six databases: MedLine (PubMed), Scopus, LILACS, IBECS, BBO, and the Cochrane Library. Additionally, the Brazilian patent database (INPI - Instituto Nacional de Propriedade Industrial) was screened in order to get an overview of Brazilian technological development in the dental materials field. Two reviewers independently analyzed the documents. Only studies and patents related to dental materials were included in this review. Data regarding the material category, dental specialty, number of documents and patents, filiation countries, and the number of citations were tabulated and analyzed in Microsoft Office Excel (Microsoft Corporation, Redmond, Washington, United States).

**Results:**

A total of 115,806 studies and 53 patents were related to dental materials and were included in this review. Brazil had 8% affiliation in studies related to dental materials, and the majority of the papers published were related to dental implants (1,137 papers), synthetic resins (681 papers), dental cements (440 papers), dental alloys (392 papers) and dental adhesives (361 papers). The Brazilian technological development with patented dental materials was smaller than the scientific production. The most patented type of material was dental alloys (11 patents), followed by dental implants (8 patents) and composite resins (7 patents).

**Conclusions:**

Dental materials science has had a substantial number of records, demonstrating an important presence in scientific and technological development of dentistry. In addition, it is important to approximate the relationship between academia and industry to expand the technological development in countries such as Brazil.

## INTRODUCTION

A wide variety of materials have been used to replace dental substrates for centuries such as animal or human teeth, bones, ivory, shells, and ceramics^4^. At the beginning, dental materials science focused on synthetic restorative materials^5^. Since then, a lot of innovations have produced new methods, devices, technologies, and products in different areas of dentistry, especially for the treatment of dental pathologies and the replacement of the dental structure^24^. Nowadays, dental materials can be classified as preventive, restorative or accessory materials^4^.

Preventive materials include pit and fissure sealants, pulp capping agents or other therapeutic agents that act as adjuvants in the control of caries progress^2^ ^,^ ^7^ ^,^ ^13^ ^,^ ^22^. Meanwhile, restorative materials are related to synthetic resins, dental adhesives systems, dental amalgam, and dental porcelain, which are used to restore the function and/or aesthetics in mouths with damaged, decayed or missing teeth^1^ ^,^ ^8^ ^,^ ^10^ ^,^ ^19^ ^,^ ^25^. Temporary restorative materials are a subcategory of restorative that include products used for dental restorations and devices that not intended to be used for moderate or long periods of time such as some dental cements (e.g., conventional glass ionomer cements or zinc oxide eugenol cement). Furthermore, accessory materials are substances or products used as adjuvants in dental procedures such as dental impression materials, dental alloys and acrylic resins^4^.

It is possible to highlight some of the most important historical events that contributed to establish concepts and techniques of current dentistry. Firstly, dental materials science was marked by the work of G.V. Black, who conducted the first controlled studies with dental amalgam in 1900 at Northwestern University^5^. The idealization of Bowen’s resin^6^ and glass ionomer cement were followed by dentin adhesive systems^16^. From that, the evolution of dentistry was driven by the development of new dental materials applied via treatments in different specialties of dentistry^5^.

In this context, the Academy of Dental Materials (ADM) was founded in 1941. It was interested in the development and application of new materials to dental care. In Brazil, the oldest group related to dental materials was the “Brazilian Group of Dental Materials” (GBMD), founded in September 29, 1965, at the “First Meeting of the Teachers of the Disciplines of Dental Materials from Dental Schools of Brazil”. For the last 50 years, GBMD aimed to provide teachers and researchers with an annual period for exchanging experiences, discussing effective ways that allow the formation of new specialized teachers, and arousing interest in the research and standardization of dental materials in Brazil. One of the group challenges is to increase the relevance of dental materials in this country, which has declined even within the undergraduate courses. As the practice of dentistry is defined by current and future developments in the science of dental materials, the aim of this study was to systematically review the literature to analyze the Brazilian scientific and technological production related to the area of dental materials over the last 50 years.

## MATERIAL AND METHODS

### Systematic literature search

The literature search was performed by two independent reviewers and involved content dating from January 1965 to December 2014. Six databases were screened: MedLine (PubMed), Scopus, LILACS, IBECS, *Biblioteca Brasileira de Odontologia* (BBO), and the Cochrane Library. Additionally, the Brazilian patent database (INPI - *Instituto Nacional de Propriedade Industrial*) was screened in order to get an overview of the Brazilian technological development in dental materials field. Furthermore, a patent search was also made using the International Patent Classification (IPC). Just as in most applications, each patent may submit more than one IPC. This technology and its applications are related to different areas of science and technology, such as dentistry, chemistry or pharmacology. The aim of identifying these codes is to create a specific tool for search and retrieval of documents.

**Figure 1:**
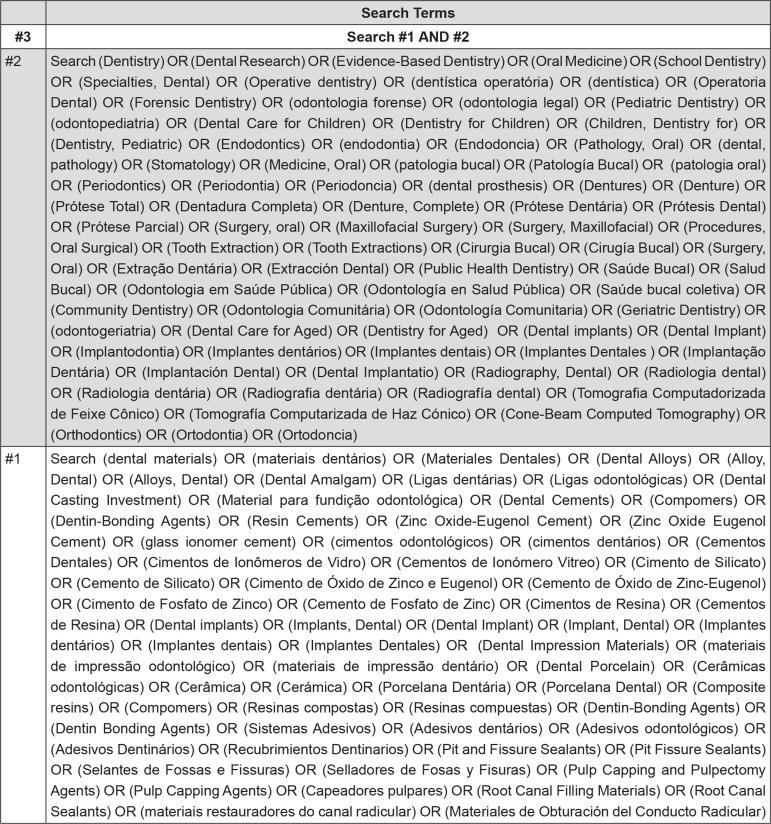
Search strategy used in PubMed (MedLine)

The keywords related to the search strategy are listed in Figure 1, which include terms related to dentistry and dental materials located in the MeSH (PubMed) and adapted for other databases. Filtering tools that were related to dental materials, as well as terms for each type of material and dental specialty, were used to optimize the search for potentially relevant papers. Endnote X7 software (Thompson Reuters, New York, New York, United States) was also used to remove duplicates. After the identification of papers in the databases related to dentistry, they were analyzed in order to include only studies of dental materials science. This systematic review is described through an adaptation of the PRISMA statement^14^.

### Study selection

Two review authors independently screened the titles and abstracts of all documents. Initially, any study related to dentistry was included. To analyze the impact of dental materials on dentistry, the inclusion criteria were: laboratory and clinical studies focusing on development/evaluation of dental materials from 1965 to 2014 and patents related to dental materials from 1965 to 2014. The exclusion criteria were the following: literature reviews, case reports, case series; studies in a language different from Portuguese, English and Spanish; and patents of dental equipment. Those appearing to meet the inclusion criteria or for which there were insufficient data in the title and abstract to make a clear decision were selected for analysis of the full document. Any disagreement regarding the eligibility of included studies was resolved through discussion and consensus or by a third reviewer.

### Data extraction and analysis

Data were extracted using filtering tools and a standardized form in Microsoft Office Excel 2013 software (Microsoft Corporation, Redmond, Washington, United States), in which the descriptive and quantitative analyses were performed. Data regarding material category, dental specialty, number of papers/patents, filiation countries, number of patents with technology transfer to companies, number of citations, and year of publication were analyzed. The material categories used were chosen from the *entry terms* found for “dental materials” in the MeSH (PubMed) database. Additionally, the Brazilian scientific production was analyzed in order to obtain an overview of the impact on the publications related to dental materials over the past 50 years.

## RESULTS

### Search strategy

**Figure 2:**
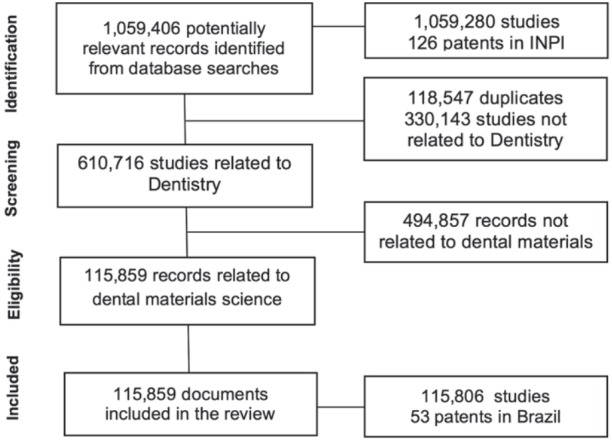
Search flowchart adapted from the PRISMA Statement

A total of 1,509,406 potentially relevant records were identified from all databases (Figure 2). After filtering and screening, 115,806 studies related to dental materials were included in this review. Regarding patents deposited by Brazil, 126 potentially relevant documents were initially screened. After screening all documents, 53 patents related to dental materials were included in this review.

### Descriptive and quantitative analysis

**Figure 3:**
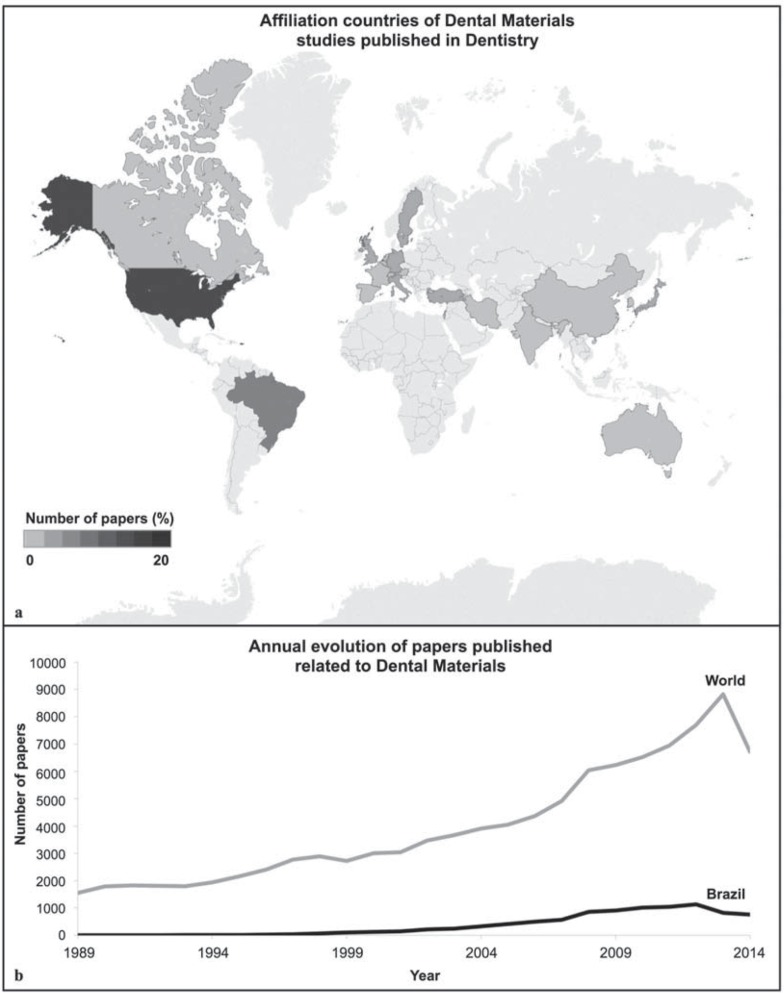
Scientific production regarding Dental Materials field: a) Studies of dental materials published according to affiliation countries (1965-2014). The majority of dental materials studies were from the United States of America (18%), followed by Brazil (8%); b) Annual evolution of papers regarding Dental Materials published (1989-2014)

Figure 3A shows that the majority of dental materials studies were from the United States of America (18%). Brazil was the affiliation country for 8% of all papers, followed by Japan (4%), the United Kingdom (4%), and Germany (4%). Figure 3B demonstrates an annual increase in published papers related to this field, with a similar increase observed in Brazil. From all papers related to dentistry, 20% represent scientific production of dental materials (1% from Brazil and 19% from the rest of the world).

**Figure 4:**
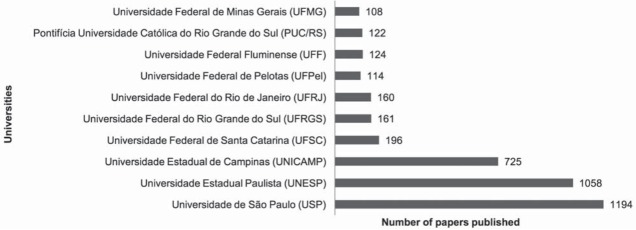
Studies of dental materials published by ten Brazilian Universities (1965-2014)

**Figure 5:**
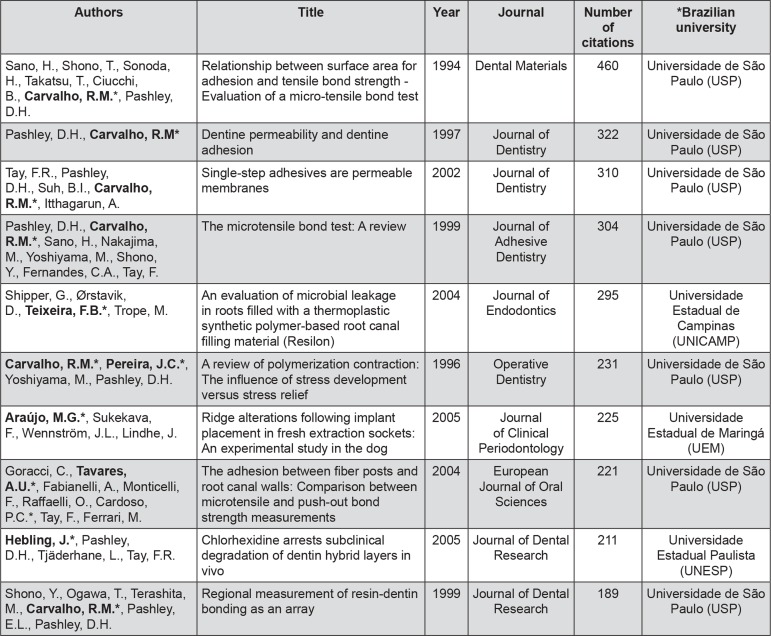
Ten most cited articles with Brazilian affiliation (1965-2014)

The Brazilian university with the most published papers was *Universidade de São Paulo* (USP), with a total of 1,194 studies, followed by *Universidade Estadual Paulista* (UNESP) with 1,058 papers, and *Universidade Estadual de Campinas* (UNICAMP) with 725 studies (Figure 4). The most-cited papers with Brazilian affiliations were from Sano, et al.^21^ (1994), with 460 citations, Pashley, et al.^16^ (1997) (322 citations), and Tay, et al.^23^ (2002) (310 citations) (Figure 5).

* Brazilian authors in bold

**Figure 6:**
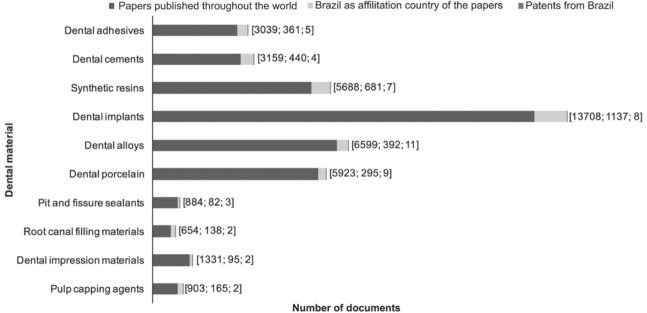
Number of papers and Brazilian patents related to dental material according to material category (1965-2014). The number between brackets represents patents published throughout the world, Brazil as affiliation country of the papers, and patents from Brazil, respectively

**Figure 7:**
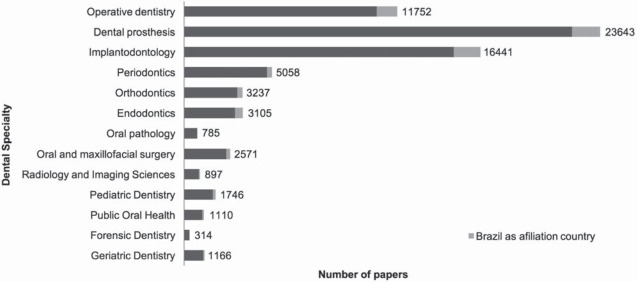
Number of papers regarding dental material published, according to dental specialty (1965-2014)

Regarding the material category (Figure 6), most papers published in the last 50 years were related to dental implants (13,708 papers), dental alloys (6,599 papers), dental porcelain (5,923 papers), and synthetic resins (5,688 papers). Furthermore, dental implants (1,137 papers), synthetic resins (681 papers), dental cements (440 papers), dental alloys (392 papers), and dental adhesives (361 papers) were the main dental materials studied in Brazil. However, the most-patented types of material were dental alloys (11 patents), followed by dental implants (8 patents) and composite resins (7 patents). Considering dental material field, the number of patents applications published in Brazil was smaller than the number of scientific papers published. Only 17% of patents deposited presented technology transfer to companies with further development of products. Besides, dental prostheses (23,634 papers), implantology (16,441 papers), and operative dentistry (11,752) were the dental specialties that most often appeared in papers about dental materials published (Figure 7).

## DISCUSSION

The main focus for dentistry will continue to be preserving and improving oral health through prevention of oral diseases, as well as the rehabilitation of lost or damaged hard and soft tissues^4^, which can be made by using synthetic materials or a tissue engineering approach. Our study demonstrated the presence of the dental materials field in different specialties. As science is in constant evolution, new techniques and materials are transforming many dental specialties in potential areas of entrepreneurship in order to create new fields of work for dentists^4^. In this context, it could be demonstrated that the publication of dental materials is not only restricted to specialized human resources, but also to several specialties such as implantology, dental prosthesis, and operative dentistry.

In addition, the results demonstrated the significant scientific participation of Brazilian production in the world scenario. With about 8%, Brazil has the second highest number of affiliations articles, ahead of more developed countries such as Japan and Germany. In this sense, the presence of laboratories with resources for physicochemical methods and the low-cost investments when compared with biological and molecular sciences can be related to the remarkable presence of studies directly or indirectly involving the investigations of dental materials. Besides, the great number of undergraduate and post-graduate students that work as part of scientific projects related to dental materials is another possible explanation. In fact, the Brazilian group was in second place in the number of papers presented at meetings of the Academy of Dental Materials (ADM) in 2014. However, it is unclear if there is a relationship between the number of published articles and studies presented in congress, meetings or conference.

It was shown that in Brazil, dental implants, synthetic resins, dental cements, dental alloys, and dental adhesives were the dental materials most studied over the last 50 years. Despite the great advances in dental materials during this period, current challenges, such as longevity of dental materials, shrinkage stress, environmental issues (e.g., Minamata protocol), and the biological effect of biomaterials (e.g., bisphenol-A release forms BisGMA-based composites), have been stimulating new projects around the world. Although significant advances occurred with synthetic materials, the best substitute for an organ or damaged tissue is the actual organ or tissue^19^. In this context, significant efforts have arisen in previous decades regarding tissue engineering, which will probably represent a progressive revolution in clinical treatments that require synthetic materials, considering that such treatments will require bioactive materials, stem cells and growth factors for regenerative approaches^18^.

Since dental materials have been shown to be important for scientific and technological development in different areas of dentistry, the concern is the decrease in the development of new tailor-made biomaterials for dentistry. However, the evolution in the Brazilian scientific production seems to have followed the annual evolution of the world scientific production in recent decades. The scientific and technological development in dentistry increases the complexity of the area and requires a professional with the competence to deal with a large number of factors, including the knowledge to work with different categories of materials that are already widely studied in Brazil (such as dental implants, synthetic resins and dental cements). Professionals with knowledge in dental materials science can magnify their labor market opportunities, as in research and development institutions, or even in dental industries, being a sector with a potential to be exploited by dentists.

Furthermore, dental materials are a science intimately linked not only to evaluating the performance of materials, but also to the development of new and better products for the dental market, which involves technologies protected by patents. The intellectual property rights protect the external exploitation of knowledge used for the development of products and innovations^3^. According to the World Intellectual Property Organization (WIPO), over 70% of technological information can only be found in the patents^20^. The “state of technique” contained in these documents provides opportunities for monitoring product licensing, drafting technology projects to obtain financing or partnerships (universities and companies) and monitoring competitors^9^ ^,^ ^20^. Patents can benefit society by the diffusion of scientific and technological knowledge, and they can protect inventors from the exploitation of the protected technology^12^.

Greater ownership of scientific information is essential to stimulate technological innovation^20^. In addition, government incentives for technological innovation are fundamental to technological development. In recent years, the “Innovation Law” (nº. 10.973/2004), a result of integration efforts between universities, research institutions and businesses, stimulated research, technological production and innovation; and the “Good Law” (nº. 11.196/2005) allowed the granting of tax incentives to companies that conduct research and technological innovation development^15^. However, in Brazil there were more scientific productions than patent deposits related to dental materials over the past 50 years, and most dental materials used by this country are foreign priorities. According to The Global Innovation Index^11^, Brazil occupied the 70^th^ position in the world ranking of innovation in 2015, behind countries in South America such as Chile (42^nd^) and Colombia (67^th^ position). In this context, it is important to stimulate innovation via research funding agencies, such as the National Council for Scientific and Technological Development (CNPq) and the Coordination of Higher Education and Graduate Training (CAPES) to promote greater incentives in areas focused on the generation and application of innovative research results. In addition, the pursuit of excellence in products and processes can stimulate the development of technological innovations not only in dental business, but also in academia^9^.

Companies have been making efforts to remain competitive through continuous innovation process. Technology push and market pull or demand pull represents the innovation strategies. Thus, companies can finance academic projects, be involved in the intellectual protection or even in the licensing of new technologies developed at the university laboratories. Furthermore, universities can still receive funds from companies as royalties to support research and innovative activities^9^. In addition, entrepreneurship can be stimulated, since universities can create joint ventures with companies, encouraging companies to hire graduate students to carry out research and development projects, or to create an environment that encourages the creation of new companies in business incubators and science parks^9^ ^,^ ^17^. Overall, it can be said that there are great opportunities for new technologies and innovative materials that could disrupt existing business models for biomaterials and supplies of traditional dentistry that could provide new treatment alternatives for patients.

Besides, it is important to highlight the current scenario of the Brazilian production being analyzed based on records retrieved from databases using MeSH Terms with other keywords in the search strategy, filtering tools and language restrictions. Most likely, several studies could not be included in pooled data because of these limitations; in addition, some patents recently deposited were not included because of an 18-month confidentiality phase. However, a high number of records were screened and analyzed in order to obtain this overview. Finally, the results suggest the need for an approximate relationship between the university and the industry to improve the integration between scientific and technological development as well as the revaluation of professional boundaries and intersections between different areas. Thus, it may be possible to increase both the scientific projection of Brazil and its representation in ownership of healthcare technologies.

## CONCLUSIONS

From this review, it was possible to obtain an overview of Brazilian production in the field of dental materials. Brazilian production demonstrated significant presence and multidisciplinary dental materials within scientific and technological scenarios of dentistry over the last 50 years. Besides, it is important to approximate the relationship between university and industry in order to stimulate continuous innovations and expand technological development in countries such as Brazil.
